# IMPROVING NURSING HOME DISASTER READINESS THROUGH IMPLEMENTATION SCIENCE

**DOI:** 10.1093/geroni/igad104.0688

**Published:** 2023-12-21

**Authors:** Sue Anne Bell, Jennifer Inloes, Michael Wasserman, John Donnelly, Tamar Wyte-Lake

**Affiliations:** University of Michigan, Ann Arbor, Michigan, United States; University of Michigan, Ann Arbor, Michigan, United States; Consultant, Santa Clarita, California, United States; VA QUERI Center for Evaluation and Implementation Resources and HSR&D Center for Clinical Management Research, Ann Arbor, Michigan, United States; U.S. Department of Veterans Affairs, North Hills, California, United States

## Abstract

The new investigation on the state of emergency preparedness in nursing homes from the Senate Finance and Aging Committee Chairs is a call to action. As large-scale disasters continue to become increasingly common worldwide, nursing homes, whose residents are more vulnerable to disaster-related health and psychosocial shocks, and their staff, are carrying progressively more responsibility for healthcare readiness practices. Implementation science is a research discipline that seeks to improve uptake of evidence-based practices, such as healthcare readiness planning, and thus has potential to improve nursing home care delivery during and after disasters. The purpose of this review was to describe the limited field of existing evidence-based strategies in the peer-reviewed literature that seek to advance healthcare readiness in the nursing home setting and illustrate how implementation science can better support healthcare readiness planning for nursing homes. We discuss three main themes: 1) implementation science frameworks can strengthen nursing home staff engagement around healthcare readiness; 2) implementation science can support tailoring of emergency preparedness plans to individual nursing homes’ unique needs; and 3) implementation science can advance the integration of nursing homes into local, state, and federal healthcare readiness planning initiatives. Finally, research is urgently needed to both generate and disseminate implementation strategies that increase uptake of evidence-based health care readiness practices in the nursing home setting.

